# Self-assembly and electrostriction of arrays and chains of hopfion particles in chiral liquid crystals

**DOI:** 10.1038/ncomms7012

**Published:** 2015-01-21

**Authors:** Paul J. Ackerman, Jao van de Lagemaat, Ivan I. Smalyukh

**Affiliations:** 1 Department of Physics, University of Colorado, Boulder, Colorado 80309, USA; 2 Department of Electrical, Computer and Energy Engineering, University of Colorado, Boulder, Colorado 80309, USA; 3 National Renewable Energy Laboratory, Golden, Colorado 80401, USA; 4 Renewable and Sustainable Energy Institute, National Renewable Energy Laboratory and University of Colorado, Boulder, Colorado 80309, USA; 5 Liquid Crystal Materials Research Center and Materials Science and Engineering Program, University of Colorado, Boulder, Colorado 80309, USA

## Abstract

Some of the most exotic condensed matter phases, such as twist grain boundary and blue phases in liquid crystals and Abrikosov phases in superconductors, contain arrays of topological defects in their ground state. Comprised of a triangular lattice of double-twist tubes of magnetization, the so-called ‘A-phase’ in chiral magnets is an example of a thermodynamically stable phase with topologically nontrivial solitonic field configurations referred to as two-dimensional skyrmions, or baby-skyrmions. Here we report that three-dimensional skyrmions in the form of double-twist tori called ‘hopfions’, or ‘torons’ when accompanied by additional self-compensating defects, self-assemble into periodic arrays and linear chains that exhibit electrostriction. In confined chiral nematic liquid crystals, this self-assembly is similar to that of liquid crystal colloids and originates from long-range elastic interactions between particle-like skyrmionic torus knots of molecular alignment field, which can be tuned from isotropic repulsive to weakly or highly anisotropic attractive by low-voltage electric fields.

## Abstract (web summary)

Topological defects can be spontaneously generated to thermodynamically stabilize a variety of peculiar condensed matter phases for technological applications. Here, Ackerman *et al*. show electrically controllable self-assembly of knotted defects into periodic arrays in chiral liquid crystals.

## Graphical abstract

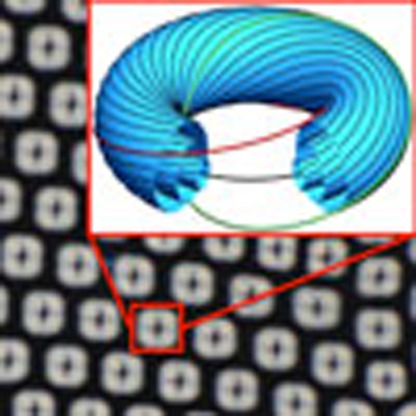

Topological defects^1^,^2^,^3^,^4^,^5^,^6^,^7^,^8^ are observed during symmetry-breaking condensed-matter phase transitions^1^ and as a result of flow^9^, application of fields^1^, temperature changes^1^,^2^,^3^ and patterning of light^10^, but typically annihilate and cannot be controlled after completion of these transient processes^1^,^2^,^3^,^9^. Rather unexpectedly, it was found recently that self-compensating topological defect pairs in active matter not only annihilate but also can be spontaneously generated, exhibiting dynamics similar to that of active particles^11^,^12^. Topological defects can form stable periodic arrays when mediating formation of thermodynamically stable vortex phases, such as cholesteric blue phases and twist grain boundary phases in liquid crystals (LCs), the A-phase of chiral magnets^4^ and Abrikosov phases of superconductors^1^,^2^,^3^. Control and generation of defects in LCs by colloids and vortex laser beams allowed for obtaining individual stable line and point defects as well as twisted solitons such as torons and localized structures resembling the mathematical Hopf fibration^5^,^6^,^7^,^8^,^13^,^14^,^15^,^16^,^17^,^18^,^19^ and even patterning of two-dimensional (2D) crystalline and quasi-crystalline arrays of such defects and solitons when pinned to confining substrates during the laser generation process^13^,^14^. To bridge these rather distinct regimes of observation of defects and topological solitons in condensed matter, we explore their field-controlled self-assembly.

Nematic LCs are three-dimensional (3D) fluids comprised of anisotropic molecules with no positional order, but with a ground state having a spatially uniform molecular long-axis orientation **n** called the ‘director’^1^. In a chiral nematic LC (CNLC), the ground-state director is twisting at a constant rate along a ‘helical axis’, with the distance over which **n**(**r**) rotates by a 2π-dubbed ‘pitch’ *p*. The CNLC ground-state twist can be suppressed by applying fields or by specially treated surfaces of confining substrates that couple to **n**(**r**), rendering it uniform and thus frustrated with respect to the twist preference. This frustration is often relieved locally through spontaneous or laser-guided formation of various cholesteric translationally invariant linear or axially symmetric solitionic structures that locally embed director twist into the unwound confined CNLCs^5^,^6^,^7^,^13^,^14^,^15^,^20^. These 2D and 3D twisted solitons resemble skyrmionic field configurations associated with Turing patterns and linked vortices in classical liquids^9^,^21^, spin textures in quantum Hall effect systems^22^, double-twisted building blocks of LC blue phases^23^,^24^ and ground states in chiral magnets^4^,^25^.

The so-called ‘double-twist cylinder’ or ‘double-twist tube’, in which **n**(**r**) is parallel to the cylinder axis at its centre and exhibits a 2D radial twist to form a barber pole-like pattern on the cylinder surface (Fig. 1a), is the basis of a 2D skyrmion that can be obtained as an isolated topological object in a confinement-unwound CNLC^26^,^27^, and is also a building block of the cholesteric blue phases with 3D crystalline arrays of such tubes^1^. The director **n**(**r**) coils around the tube with periodicity defined by the distance along the tube axis over which **n**(**r**) winds around it once (Fig. 1a). When a fragment of such a tube of length corresponding to a single winding of **n**(**r**) is looped on itself, the director field lines of the ensuing 3D skyrmion in the form of a double-twist torus become closed circles linked with each other (Fig. 1a, top right)^5^, with single windings around the circle in the interior of the torus (*P*=1) and also around its axis of rotational symmetry (*Q*=1) (Fig. 1b)^28^,^29^. The linking of circles of **n**(**r**)-loops in this 3D skyrmionic structure resembles that of ‘fibres’ in the famous mathematical Hopf fibration^3^,^5^,^30^,^31^. Similar construction through looping different 2D skyrmions results in double-twist tori with multiple windings around the two axes and the mutually interlinked (*P*,*Q*) torus knots in **n**(**r**), resembling a more general class of mathematical Seifert fibrations^29^,^30^,^31^, which include the Hopf fibration in a limited case of *P*=*Q*=1 (ref. 5). An example of a 3D skyrmion formed by a double-twist torus with linked (*P*,*Q*)=(2,3) trefoil knots is shown in the bottom-right of Fig. 1a, with two representative mutually linked knots of **n**(**r**)-loops shown in red and green colours. The 3D skyrmions with such (*P*,*Q*) knots are characterized by a non-zero Hopf charge *C*_H_=*PQ* and are commonly called ‘hopfions’^29^. They are theoretically predicted to exist in many fields of science^28^,^29^,^30^,^31^,^32^,^33^,^34^,^35^,^36^,^37^, but convincing experimental demonstrations of their stability and direct detailed explorations of their structure remain elusive^29^. Since the nematic ground-state manifold **R***P*^2^ is a sphere with antipodal points identified, these 3D skyrmions in CNLCs are labelled by elements of the third homotopy group^6^,^26^, *π*_3_(**R***P*^2^)=**Z**. It is remarkable that CNLCs can host such skyrmions both as separate field configurations and when accompanied by various other defects and colloids^5^,^6^,^7^,^13^,^14^,^15^. For example, these skyrmions form the basis of torons^5^ in which the double-twist tori hosting (*P*,*Q*) knots of **n**(**r**) are accompanied by two self-compensating point or ring-shaped defects (Fig. 1c,d)^5^,^6^,^7^, often appearing to reduce the elastic and surface-anchoring free energies associated with matching the **n**(**r**) of such solitonic structures with the far-field director and weak or strong boundary conditions on confining surfaces of CNLC cells. The continuous knotted **n**(**r**) of the 3D skyrmions cannot be unknotted without energetically prohibitive ‘cutting’ of the **n**(**r**)-director field lines, giving rise to particle-like properties.

In this work, we demonstrate that these topologically protected skyrmionic particles self-assemble into various crystalline arrays and linear chains. The facile frequency-dependent electric response of CNLCs enables large-quantity generation, guided self-assembly and electrostriction of dense and sparse periodic arrays and linear chains of skyrmions, which are often accompanied by various other singular and nonsingular topological defects. Being topologically protected field configurations, with mutually linked different torus knots of looped molecular alignment field lines, these particle-like solitons self-assemble similar to LC colloids and electrostatic dipoles^8^. Interactions between the skyrmions are electrically switched between repulsive and attractive, mediating self-assembly of crystalline arrays and chains with tunable inter-particle spacing. The exquisite control of topologically nontrivial structures is achieved at voltages of the order of 1 V, potentially enabling mesostructured soft-matter composites with tunable optical properties and a host of new technological applications.

## Results

### Facile generation and structure of skyrmionic particles

**Figure 1:**
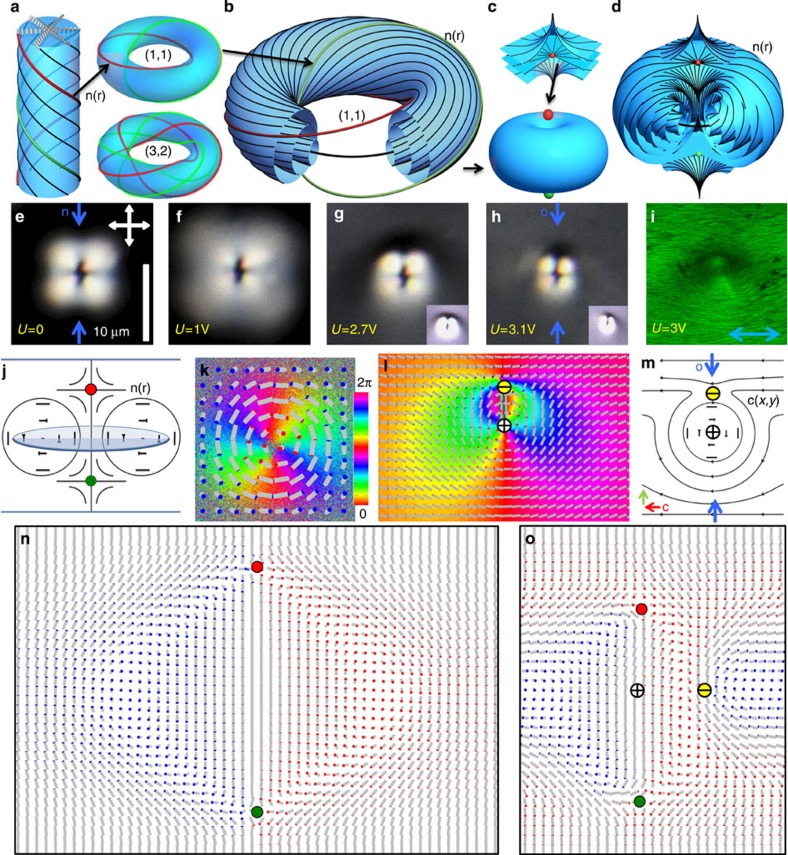
Structure and topology of 3D skyrmionic particles. (**a**) Double-twist cylinder of **n**(**r**) and its use in the construction of tori with linked (*P*,*Q*)=(1,1) unknots (top right) and various torus knots, such as the (*P*,*Q*)=(3,2) trefoil knots (bottom right); the green and red **n**(**r**) lines are used for eye guiding to depict linked unknots and knots of **n**(**r**) loops. (**b**) A double-twist torus formed by a double-twist cylinder looped on itself is a 3D skyrmion-dubbed ‘hopfion’. (**c**) When accompanied by two self-compensating hyperbolic point defects of opposite hedgehog charges ±1 (shown in red and green colours), the hopfion forms a toron. (**d**) **n**(**r**) structure of the toron. (**e**–**h**) Polarizing optical micrographs of a toron between two crossed polarizers (shown by white double arrows) at *U* marked on the images. The toron is surrounded by a uniform unwound vertical **n**(**r**) at low *U*, but becomes embedded in the TIC above *U*≈2.1 V threshold voltage, which is slightly dependent on *d*/*p*. The insets in **g** and **h** were obtained for illumination settings chosen to highlight the dipolar structure of **c**(*x*,*y*) and the umbilic defect. (**i**) 3PEF-PM image of a polymerized CNLC sample revealing **c**(*x*,*y*) of the toron–umbilic pair. The far-field orientations of the midplane **n**(**r**) and **c**(*x*,*y*) is denoted by a blue double arrow. (**j**) Schematic of **n**(**r**) in the vertical cross-section of an axially symetric toron, with the plane of images (**i**,**k**) shown in blue colour. (**k**) Experimentally reconstructed colour-coded pattern of azimuthal orientation of **n**(**r**) in the XY cross-section in the midplane of a toron at *U*=0 co-located with the numerically simulated **n**(**r**) shown using cylinders with red-blue-coloured ends; the colour scheme is shown in the inset. (**l**) Computer-simulated **n**(**r**) shown using cylinders and its azimuthal orientation depicted using colours for a toron embedded in TIC in the cell midplane. The white and yellow circles with ‘±’ depict signs of winding numbers of the ±1 defects in **c**(*x*,*y*). (**m**) The corresponding qualitative schematic of the **c**(*x*,*y*). (**n**,**o**) **n**(**r**) of the topological particles shown in the vertical cross-sections marked on micrographs (**e**,**h**) at corresponding (**n**) *U*=0 and (**o**) *U*=3.1 V. Green- and red-filled circles depict hyperbolic point defects with hedgehog charges ±1. The two ±1 singular points in **c**(*x*,*y*) shown in **o** are nonsingular in **n**(**r**) and correspond to the parts of the cell midplane with vertical **n**(**r**) marked by white and yellow circles.

Skyrmions with different torus knots of **n**(**r**) can be generated in confinement-unwound LC cells on an individual basis by focused beams or colloidal inclusions^5^,^6^,^7^, as in the example of a laser-generated toron shown in Fig. 1e. In addition to linked unknots (*P*,*Q*)=(1,1), trefoil and pentafoil torus knots of **n**(**r**) also can be observed within the double-twist-torus part of the toron structures. This structural behaviour is highly dependent on the details of toron generation, sample thickness over pitch ratio *d*/*p*, lateral confinement and other factors that have been detailed previously^5^,^6^,^7^,^13^,^14^,^15^ as well as will be explored further elsewhere, but, importantly for the present work, all of these skyrmionic configurations exhibit particle-like behaviour. Such skyrmionic configurations are stable long after generation, but can be electrically controlled by applying voltages *U*=(1–4) V at 1 kHz (Fig. 1e–h). Consistent with the fact that the used LCs have negative dielectric anisotropy *Δε*, the difference between dielectric constants measured for electric field along and perpendicular to **n**, the toron structure first laterally expands with increasing *U* (Fig. 1e,f), which is expected as its interior has in-plane orientation of **n** favoured by the free-energy term describing its coupling to the field. However, as *U* increases further, prompting a transition of the initially vertical director **n** to in-plane **n**(**r**) with twist across the cell thickness accompanied by bend/splay distortions^20^, the effective lateral size of the toron starts to shrink and the birefringent dipolar-like texture around it becomes visible between crossed polarizers (Fig. 1f–h). This is consistent with depth-resolved three-photon excitation fluorescence polarizing microscopy (3PEF-PM)^6^,^7^,^38^,^39^ (Fig. 1i,k) and numerical modelling of such structures at various *U* (Fig. 1k–o). To understand the physical underpinnings, it is instructive to consider the midplane of a cell passing through the equatorial plane of a toron (marked by a blue disc in the simplified structural model shown in Fig. 1j). Figure 1k depicts an experimentally reconstructed pattern of azimuthal orientation of **n**(**r**) in this plane at *U*=0, overlaid with the corresponding numerically simulated **n**(**r**), which we show using cylinders with blue/red ends. The **n**(**r**)-tilt direction at large *U*, which can be described by a 2D vector field **c**(*x*,*y*) decorating the projection of **n**(**r**) onto the cell midplane and is concentric at the periphery of a toron, must match the uniform far-field **c** (Fig. 1l,m). This is achieved by introducing a defect in **c**(*x*,*y*) with a winding number −1, known as ‘umbilic’^1^, that compensates for the +1 defect in **c**(*x*,*y*) in the toron centre. The **c**(*x*,*y*) of the ensuing toron–umbilical topological dipole is shown in Fig. 1m, with the dipole marked by a green arrow.

**Figure 2:**
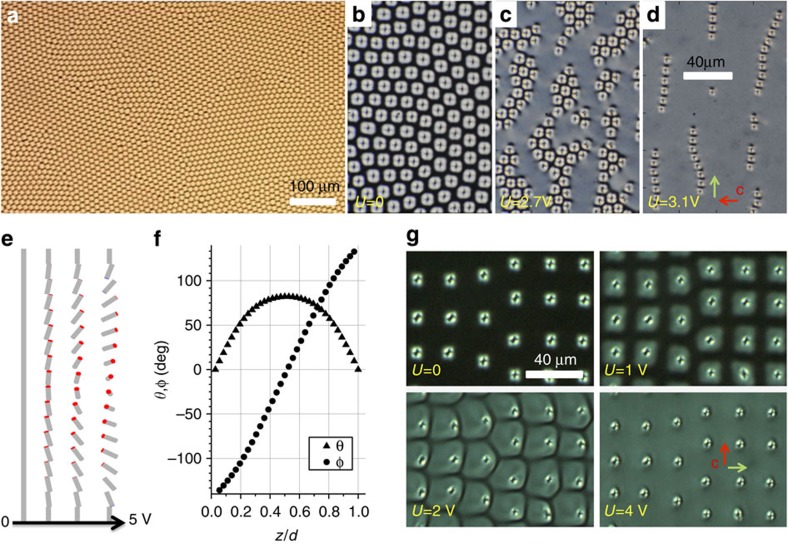
Elastic self-assembly and patterning of topological particles. (**a**) An optical micrograph showing hexagonal ordering in dense arrays of torons, with large-area crystallites separated by grain boundaries. (**b**–**d**) Polarizing optical micrographs depicting voltage-controlled transitions from (**b**) isotropic repulsive interactions mediating formation of hexagonal arrays to (**c**) weakly anisotropic attractive interactions resulting in crystallites of topological particles with smaller periodicity, and (**d**) to highly anisotropic interactions that result in chains of toron–umbilical dipoles. The red arrow in **d** denotes the orientation of the far-field **c** and the green arrow depicts orientation of the toron–umbilical dipoles. (**e**) Computer-simulated **n**(**r**), depicted using cylinders, across the sample without topological particles at different *U*. (**f**) The corresponding polar (*θ*) and azimuthal (*φ*) orientation angles of **n**(**r**) at *U*=3 V. (**g**) Polarizing optical micrographs showing substrate-pinned laser-generated torons in an array with a deliberately introduced edge dislocation at different *U*; note that the surface-pinning prevents elastic interactions between these pinned topological particles, but exhibits transformation of **n**(**r**) and dipolar configurations in **c**(*x*,*y*) versus *U* similar to that of their ‘mobile’ counterparts.

In CNLCs doped with ionic surfactants^40^, large quantities of torons can be generated through the relaxation of the frustrated-state-confined CNLC from hydrodynamic instability (Supplementary Fig. 1). Upon turning off the field, the CNLC relaxes from the disordered hydrodynamic state to a ground-state configuration with varied density of torons that self-organize into periodic hexagonal arrays (Fig. 2a), consistent with our numerical findings that arrays of torons correspond to the ground state of confined CNLCs at cell thickness *d* to pitch ratio *d*/*p*≈1 (ref. 5). The hydrodynamic instability plays the role of generating torons in the entire sample similar to how focused laser beams do this locally^5^,^6^,^7^, that is, it allows the system to relieve frustration imposed by boundary conditions via formation of toron arrays (Fig. 2a,b). When generated at varying densities, toron particles form Wigner-crystal-like hexagonal lattices due to isotropic repulsive interactions (Fig. 2a,b; Supplementary Fig. 2). The 2D crystallites with hexagonal arrangements of torons are hundreds of micrometres or even millimetres in size, 100–1,000 times larger than individual torons (Fig. 2a). Uncontrolled orientation of crystallographic axes of the crystallites upon their formation through relaxation from the hydrodynamic instability causes grain boundaries (Fig. 2a).

### Electric switching and self-assembly

**Figure 3:**
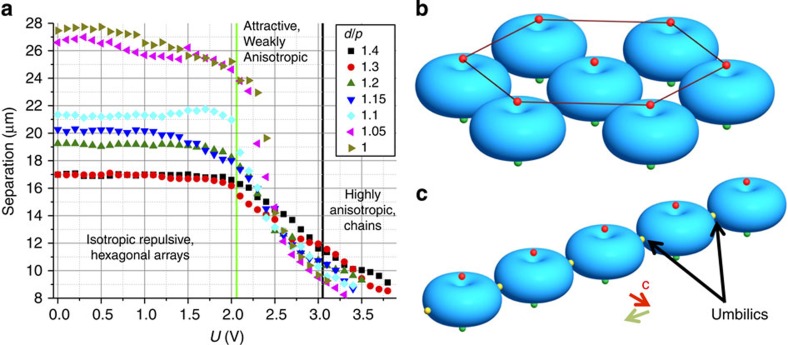
Electric control of interactions and assembly of topological particles. (**a**) Inter-particle separation in equilibrium structures versus *U* at different *d*/*p*. The green line marks the homeotropic-TIC transition. The black line marks the transition between hexagonally arranged attractive torons and linear chains of toron–umbilical dipoles. (**b**) A schematic of hexagonal assembly of topological particles. (**c**) A schematic of dipolar chains of torons inter-spaced by umbilical defects (depicted as yellow spheres) in the **c**(*x*,*y*).

Application of electric fields above a threshold voltage of *U*≈2.1 V at 1 kHz leads to a transition of the unwound homeotropic **n**(**r**) to the so-called ‘translationally invariant configuration’ (TIC) of **n**(**r**)^20^, with a voltage-dependent twist and bend-splay distortions across the cell thickness (Fig. 2e,f). With increasing *U*, similar to individual topological particles (Fig. 1e–h), lateral dimensions of torons within the arrays first expand and then shrink while inducing the umbilical defects in **c**(*x*,*y*). This leads to the reversal of interactions from repulsive to attractive, self-assembly of torons into hexagonal lattices of voltage-dependent periodicity, and finally to the formation of chains of toron–umbilical dipoles (Fig. 2c,d; Supplementary Fig. 2), as we summarize in Fig. 3. The dipolar chains (Figs 2d and 3c) resemble the ones formed by electrostatic dipoles and elastic dipoles in nematic LC colloids^8^, except that our topological defect dipoles always have the same orientation orthogonal to **c** because of the polar nature of **c**(*x*,*y*) (Fig. 2d). Similar to the case of nematic colloids, both repulsive and attractive interactions between the topological particles occur to minimize the free energy as the corresponding director configurations transform in response to fields. Finally, at *U*>5 V, dipolar defect chains become fully unstable due to the strong coupling between the electric field and **n**(**r**) in the LC with negative dielectric anisotropy, which discontinuously destroys the topological particles. Annihilation of torons and umbilics, which includes mutual destruction of the hopfion-like double-twist torus structure and umbilic, and annihilation of oppositely charged hyperbolic point defects, eventually lead to a uniform TIC. Within the stability range of the skyrmionic particles, the inter-particle spacing changes markedly by a factor of 2–3 with varying *U* (Fig. 3a), yielding a giant electrostriction of 2D hexagonal and linear chain assemblies that shrink by over 50% at 0.5 V μm^−1^. The voltage-tunable self-assembly of particle-like ‘mobile’ torons is markedly different from that of torons pinned to the confining surfaces (Fig. 2g) optically generated at high laser powers above 70 mW. In the latter case, upon increasing *U* above the threshold for inducing TIC, the torons remain pinned to the spatial locations of their generation despite the formation of toron–umbilical dipoles (Fig. 2g), until disappearing at ~5 V.

### Dynamics and characterization of pair interactions

**Figure 4:**
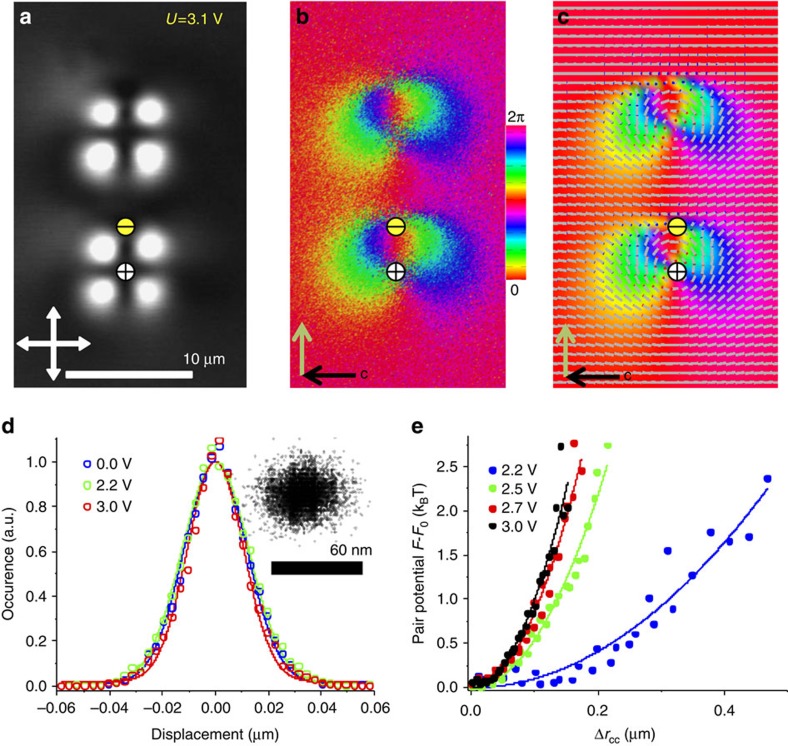
Structure and dynamics of a dimer of toron–umbilical dipolar particles. (**a**) Polarizing optical micrograph of a toron–umbilical pair embedded in the TIC at *U*=3.1 V; white double arrows depict orientations of crossed polarizers. (**b**) The corresponding experimentally reconstructed pattern of azimuthal orientation of **n**(**r**) in the cell midplane presented using the colour scheme shown in the inset. (**c**) The corresponding computer-simulated **n**(**r**) shown using cylinders, which is overlaid atop of the pattern of azimuthal orientations of **n**(**r**) presented in the same way as the experimental one shown in **b**. The white and yellow circles with ‘±’ marks in (**a**–**c**) depict signs of winding numbers of the ±1 defects in **c**(*x*,*y*). (**d**) The histograms of a single topological particle displacement at different *U* reconstructed using videomicroscopy. (**e**) The relative pair potential energy *F−F*_0_ versus displacement from the equilibrium centre-to-centre inter-particle separation at different *U* reconstructed by probing thermal fluctuations of the inert-particle distance using videomicroscopy.

Skyrmionic topological particles and their self-assemblies undergo Brownian motion (Fig. 4), which we characterize using videomicroscopy by probing their displacements during time intervals *τ*=200 ms over 18.3 min. This highly overdamped motion (Reynolds number ≪1) is direction independent when a toron is embedded in the unwound CNLC, but becomes slightly anisotropic in TIC at *U*=(2.1–5)V. The direction-averaged half-width *Δ* of histograms of displacements described by Gaussian distributions (Fig. 4d) yield voltage-dependent diffusivity^1^ of the topological particles within *D*=*Δ*^*2*^/*τ*=(1.1–1.6) × 10^−3^  m^2^ s^−1^, which was found to be independent of frequency within the 1–10 kHz used to electrically guide self-assembly. Using this experimental diffusion constant, we find the effective viscous drag coefficients from the Einstein relation *ζ*=*k*_*B*_*T*/*D*=(2.6–3.7) × 10^−6^ Ns m^−1^, where *k*_*B*_=1.38 × 10^−23^ J K^−1^ is Boltzmann’s constant and *T* is temperature^1^,^2^. The forces arising from minimization of free energy pull the toron–umbilical dipoles to an equilibrium distance (Fig. 4a–d) and lead to *U*-dependent histograms of their relative centre-to-centre separations *P*(*Δr*_*cc*_) away from the equilibrium. The relative pair-interaction free energy *F−F*_0_ near the equilibrium state with free energy *F*_*0*_ (Fig. 4e) is then computed from this experimental histogram by inverting the Boltzmann relation, *P*(*Δr*_*cc*_)∝exp (*F−F*_0_)/*k*_*B*_*T*. As the equilibrium inter-particle separation decreases with increasing U upon formation of chains (Fig. 3a), the spring constant *k* describing Hookean-like behaviour near the equilibrium *F−F*_0_=*kΔr*_*cc*_^*2*^ increases with voltage within *k*=0.08–0.83 pN *μ*m^−1^ (Fig. 4e).

**Figure 5:**
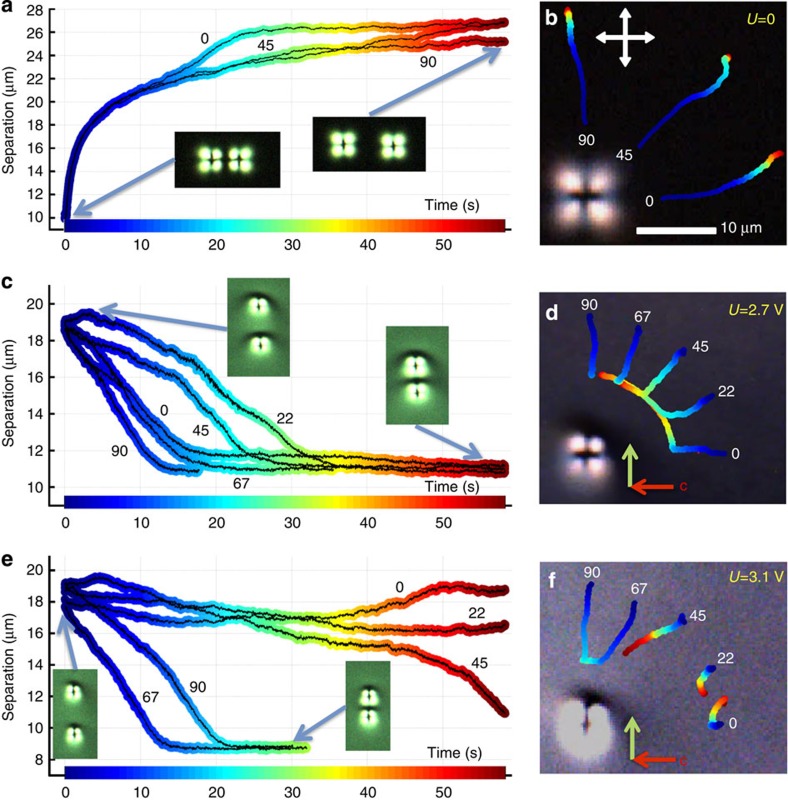
Pair interactions of topological particles. (**a**) Colour-coded centre-to-centre separation vs time trajectories for two particles released with initial separation vectors at 0, 45 and 90 degrees with respect to the far-field **c** demonstrating isotropic inter-particle repulsion at *U*=0. (**b**) The corresponding colour-coded-time trajectories of one toron of the pair with respect to the position of the second. White double arrows show orientations of crossed polarizers. (**c**–**f**) Centre-to-centre separation and trajectories similar to the ones shown in **a** and **b**, but at (**c**,**d**) *U*=2.7 V and (**e**,**f**) *U*=3.1 V; the initial angles between the inter-particle separation vectors and the far-field **c** are 0, 22, 45, 67 and 90 degrees, as marked next to the trajectories. Red arrows in **d** and **f** denote the far-field **c** and the green arrows denote the orientation of the toron–umbilical dipoles.

To uncover physical underpinnings behind self-assembly of skyrmionic particles, we use holographic laser tweezers^28^ capable of trapping and manipulating them at different *U*. Topological particles can be placed at desired initial positions corresponding to different inter-particle separation vector orientations with respect to the far-field **c** (Fig. 5). Once released from the traps, topological particles repel or attract, depending on the initial conditions and *U*, allowing us to probe both pair interactions (Fig. 5) and many-body interactions (Supplementary Fig. 3), which we study using videomicroscopy. As voltage increases, the interactions change from isotropic repulsive (Fig. 5a,b) to isotropic and then weakly anisotropic attractive (Fig. 5c,d), and finally to strongly anisotropic dipolar-like (Fig. 5e,f), with the parallel toron–umbilical dipoles attracting when their centre-to-centre separation vector is orthogonal to the far-field **c** (Fig. 5f; Supplementary Movie 1) and repelling when it is parallel to the far-field **c** (Fig. 5f; Supplementary Movie 2). Remarkably, this marked change occurs as *U* is varied within a narrow range of (0–3.5) V, corresponding to the self-assembly of larger- and smaller-period hexagonal lattices and dipolar chains of toron–umbilical dipoles aligned orthogonal to the far-field **c** (Figs 2 and 3). From the topological particle position versus time data, we calculate their velocities that range within d*r*/d*t*=(0–3) μm s^−1^. Neglecting inertia effects, we estimate the inter-particle elasticity-mediated forces from their balance with the effective viscous drag force −*ζ*d*r*/d*t*=(0–10) pN, consistent with their origin as the values of elastic constants are of the order of 10 pN (Supplementary Table 1).

### Comparison of experiments and numerical modelling

Our experimental findings are fully reproduced by numerical modelling based on the director relaxation method^5^,^29^ of minimizing the CNLC free energy at strong perpendicular boundary conditions on confining plates^1^. Since the half-integer defect lines in **n**(**r**) do not occur in the structures studied in this work, this approach is ideally suitable for 3D modelling of the skyrmionic particles as it accounts for all elastic constants while also allowing for simulations of relatively large sample sizes ranging from micrometres to tens of micrometres (computer simulations accounting for elastic anisotropy based on the Q-tensor approach for such large samples would be rather slow)^5^,^29^. Taking experimental material and geometric parameters, we obtain the equilibrium 3D **n**(**r**) configurations of torons and toron–umbilic dipoles embedded in the TIC at various voltages (Figs 1k,l,n,o and 2e,f; Supplementary Fig. 4) closely matching their experimental counterparts. Modelling of elastic pair interactions of these particles also closely reproduces experimental results. For example, Fig. 4b,c shows the experimentally reconstructed (from 3PEF-PM images) and corresponding computer-simulated colour-coded **n**(**r**) configurations in the equatorial plane of the toron–umbilic dipole passing through the cell midplane; the simulated **n**(**r**) is additionally represented using cylinders with coloured ends overlaid on the top of the colour-coded texture (Fig. 4c). This agreement between modelling and experiments supports our explanation of self-assembly of skyrmionic particles through the electrostatic and nematic colloidal analogies^8^.

## Discussion

On the basis of both numerical modelling and experiments, tunable elastic interactions between skyrmionic topological particles can be qualitatively explained as follows. At no applied fields, the **n**(**r**) structure of a toron embedded into a uniform far-field director (Fig. 1d) has quadrupolar symmetry. Therefore, the torons interact repulsively as elastic quadrupoles (Fig. 4a,b), although these interactions in lateral directions are screened by confinement of the CNLC into a cell with strong boundary conditions. As the lateral spatial extent of **n**(**r**) distortions smoothly increases with *U* at voltages below the realignment threshold away from torons, the strength of their quadrupole moments and lateral distance range of repulsive interactions increase too. At the realignment threshold, a symmetry-breaking structural transition occurs, transforming the initial quadrupolar toron into an elastic toron–umbilical dipole with an in-plane orientation of the dipiole moment orthogonal to the far-field **c**. As the strength of the elastic dipole moment gradually increases with *U*, the elastic interactions gradually transform from isotropic quadrupolar repulsive at low *U* to strongly anisotropic dipolar-like attractive at relatively high *U*, giving the origin to different voltage-dependent self-assemblies. Beyond this qualitative picture, quantitative understanding of interactions between skyrmionic particles requires accounting for detailed contributions of twist and dielectric terms associated with the complex 3D structure of the toron–umbilical field configurations at different fields, as well as the short-range interaction effects that cannot be described through the electrostatic analogy, further contributing to the richness and complexity of interactions in cholesteric systems that recently attracted a great deal of attention^41^.

Another interesting feature of our system is the diversity of different classes of topological defects co-existing with each other. The *π*_3_(**R***P*^2^) hopfions in the central part of the toron are accompanied by self-compensating *π*_2_(**R***P*^2^) point defects of opposite hedgehog charges, which could be replaced by loops of *π*_1_(**R***P*^2^) line defects when generating torons by optical vortices^5^, and also by nonsingular in **n**(**r**) umbilical defects, which are singular point defects *π*_1_(*S*^1^) in the 2D **c**(*x*,*y*) field. The stability of these defects of different topological classes while being assembled into various voltage-tunable periodic arrays and chains may allow for using our system in probing details of their structure and interactions, which may provide new insights into the topological nature and properties of similar skyrmionic field configurations predicted to exist in other physical systems^28^,^29^,^30^,^31^,^32^,^33^,^34^,^35^,^36^,^37^,^42^,^43^,^44^. From the standpoint of view of practical applications, because of the contrast of the effective refractive index between the twisted and non-twisted regions of periodic arrays^14^, the self-assembled structures of skyrmionic particles can be readily used as electrically reconfigurable diffraction gratings (Supplementary Fig. 5) and optical vortex generators^13^,^45^. Giant electrostriction, which we find to be even stronger than that observed in LC colloids^46^, light sensitivity, which could be further enhanced by doping the LC with dyes and using other types of generating light^47^ and entrapment of metal and semiconductor nanoparticles within these topological particles that we demonstrated recently^15^ can be used in combination to form composites with new tunable properties emerging from controlling mesoscopic order of the nanoparticles.

To conclude, we have described facile generation and voltage-tunable self-assembly of 3D skyrmions, along with different types of singular and nonsingular defects, into periodic arrays and linear chains emerging from reconfigurable elasticity-mediated interactions. Furthermore, the inter-skyrmion separation in the self-organized structures was tuned by varying applied voltages up to 5 V, giving rise to strong electrostriction. This behaviour bridges markedly different forms of observation of condensed matter defects, ranging from active LCs to thermodynamically stable phases with periodic vortex lattices. The exquisite control of self-assembly of skyrmionic field configurations and singular topological defects may enable their practical uses in diffractive optical elements, singular optics, nanoparticle entrapment into periodic arrays and in fabrication of mesostructured composites.

## Methods

### Sample preparation and characterization

The CNLC was prepared by mixing negative dielectric anisotropy nematic host ZLI2806 and chiral additive CB15 with the helical twisting power *H*_HTP_=6.1 *μ*m^−1^ (both from EM Chemicals). The equilibrium pitch *p*=1/(*H*_HTP_ × *C*_a_) was varied within 7–20 *μ*m by tuning the concentration *C*_a_ of the additive. The CNLC was doped with~0.1wt.% of cationic surfactant Hexadecyltrimethylammonium bromide (CTAB, from Sigma-Aldrich) to promote hydrodynamic instability when applying low-frequency fields. Cells were constructed from glass substrates with transparent indium tin oxide electrodes treated for vertical surface boundary conditions by dip coating in an aqueous solution of 10 mg ml^−1^ of CTAB. The cell gap was varied within 7–15 *μ*m and was set by glass spacers dispersed in ultraviolet-curable glue. The dihedral angle between cell substrates was kept below two degrees, such that the local thickness variation can be neglected. Constructed cells were infiltrated by the CNLC (while heated to isotropic phase to avoid the flow effects on alignment) by means of capillary forces and then sealed with 5-min epoxy. Voltage waveforms were produced by a DS345 generator (Stanford Research Systems) and applied using wires soldered to the transparent electrodes. Dense arrays of torons (Fig. 2a,b) were generated by applying a 1-Hz square wave at 10 V peak to peak for ~10 s. This caused hydrodynamic instability^1^,^40^ that was then relaxed to arrays of torons (Supplementary Fig. 1). In experiments involving the control and self-assembly of skyrmionic particles, a 1-kHz square wave of varied *U* was used to avoid the hydrodynamic turbulence needed only for toron generation. Torons were also generated on individual basis using holographic optical tweezers, both as surface-pinned (at laser powers 70–150 mW, Fig. 2g) and mobile (at laser powers 30–50 mW, Fig. 1) topological particles; the tweezers were additionally used for non-contact manipulation. The experimental reconstruction of 3D director fields was performed by use of multimodal nonlinear optical polarizing microscopy, built around an inverted microscope (Olympus IX81) and integrated with laser tweezers^6^,^14^,^15^,^16^,^26^. Both 3PEF-PM and two-photon excitation fluorescence polarizing microscopy (2PEF-PM) were used, as described in details in the Supplementary Material and elsewhere^26^,^27^,^28^.

### Approaches and implementation of numerical modelling

Assuming infinitely strong homeotropic surface anchoring at confining plates, the 3D equilibrium **n**(**r**)-structures at different electric fields **E** were studied through the minimization of the bulk free energy^1^,^48^ *F*=*F*_elastic_+*F*_electric_, with 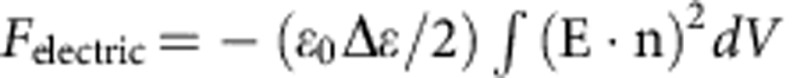 describing the coupling of **E** and **n**(**r**) and Frank–Oseen elastic energy given by


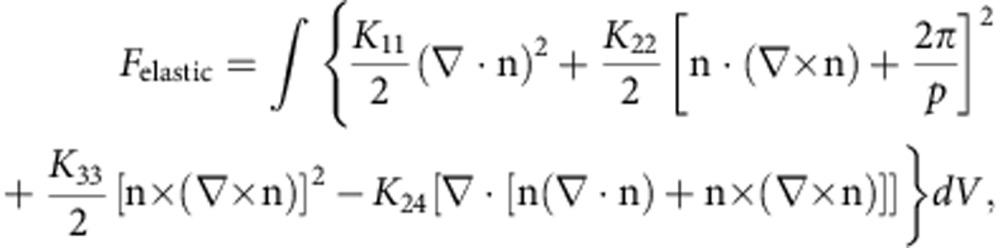


where *K*_11_, *K*_22_, *K*_33_ and *K*_24_ are elastic constants for splay, twist, bend and saddle splay deformations, respectively. Using experimental material parameters (Supplementary Table 1) and a 119 × 119 × 35-rectangular grid, we have implemented a recursive relaxation routine that minimizes *F* with a finite difference method applied to the internal grid points^48^. The steady state is determined through monitoring changes of the spatially averaged functional derivative given by the Lagrange equation. When these changes asymptotically approach zero, the system is assumed to be in a ground state, yielding 3D **n**(**r**) structures such as the ones shown in Fig. 1n,o. A detailed description of this method is provided in the Supplementary Material.

### Computer simulations of polarizing optical micrographs

On the basis of the computer-simulated 3D director structure of our topological particles, we have simulated the corresponding polarizing optical micrographs using the Jones matrix method^49^,^50^ and experimental material parameters such as optical anisotropy, pitch and cell thickness. The LC sample with a topological particle was split into a set of thin slabs parallel to substrates with known orientation of **n**(**r**) given by numerical modelling described above. While traversing through the LC cell, light splits into ordinary and extraordinary waves with electric fields perpendicular and parallel to the in-plane projection of **n**(**r**), respectively. The effect of each thin slab is equivalent to that of a phase retardation plate with a spatially varying optical axis, and is described by a coordinate-dependent Jones matrix^49^,^50^. In each pixel of a simulated polarizing microscopy texture, intensity of the light after propagation through the cell is obtained by successive multiplication of the Jones matrices corresponding to a polarizer, the series of thin CNLC slabs with coordinate-dependent phase retardation and the analyzer. To mimic the achromatic-light observations in the experiments, we have performed these calculations for wavelength 475, 510 and 650 nm and then superimposed the resulting textures to obtain the polarizing optical micrographs. Computer-simulated images (Supplementary Fig. 4) closely resemble experimental results (Fig. 1e–h) and further reaffirm our understanding of director configurations based on computer simulations and reconstruction using 3PEF-PM images.

## Author contributions

P.J.A. and I.I.S. performed experimental work. P.J.A., J.v.d.L. and I.I.S. analysed experimental results. P.J.A. did numerical modelling. P.J.A. and I.I.S. reconstructed director fields and defect structures. J.v.d.L. and I.I.S. provided funding. P.J.A. and I.I.S. wrote the manuscript. I.I.S. conceived and designed the project.

## Additional information

**How to cite this article:** Ackerman, P. J. *et al*. Self-assembly and electrostriction of arrays and chains of hopfion particles in chiral liquid crystals. *Nat. Commun.* 6:6012 doi: 10.1038/ncomms7012 (2015).

## Supplementary Material

Supplementary Information Supplementary Figures 1-5, Supplementary Table 1, Supplementary Methods and Supplementary References

Supplementary Movie 1 Attractive pair-interaction of two parallel toron-umbilical dipoles observed when their center-to-center separation vector is oriented perpendicular to the far-field c, with both dipoles being orthogonal to it as well.

Supplementary Movie 2 Repulsive pair-interaction of two parallel toron-umbilical dipoles observed when their center-to-center separation vector is oriented parallel to the far-field c, with both dipoles being orthogonal to it.
