# Supplementary material for: Self-assembly and electrostriction of arrays and chains of hopfion particles in chiral liquid crystals
Source: Nat Commun. 2015 Jan 21;6:6012. doi: 10.1038/ncomms7012 (PMC4354077; doi:10.1038/ncomms7012)
Supplement: Supplementary Information — Supplementary Figures 1-5, Supplementary Table 1, Supplementary Methods and Supplementary References [file ncomms7012-s1.pdf]

Supplementary Figures

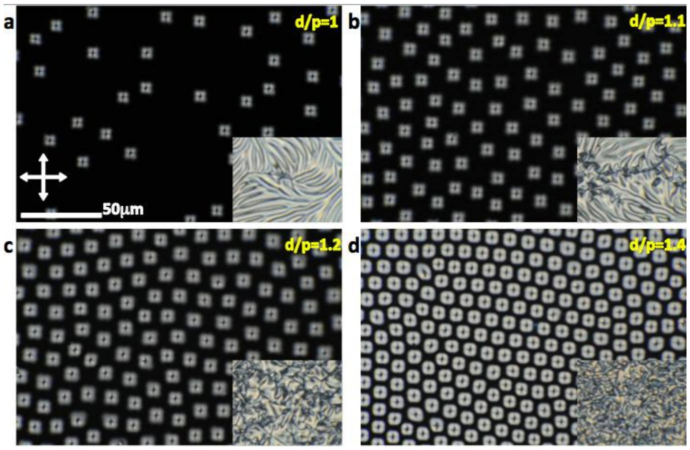

Supplementary Figure 1 | Generation of torons and their arrays through relaxation from the hydrodynamic instability. a-**d**, Polarizing optical micrographs of torons generated through relaxation of hydrodynamic instabilities induced by applying a 1Hz square wave at *U*=10 V peak to peak for approximately 10 s. The insets show polarizing optical micrographs of the sample during the hydrodynamic turbulence. White double arrows mark the orientation of the crossed polarizer and analyzer. As *d*/*p* ratio (marked on the images) increases, the density of torons increases too, leading to lateral confinement and crystallization due to repulsive interactions.

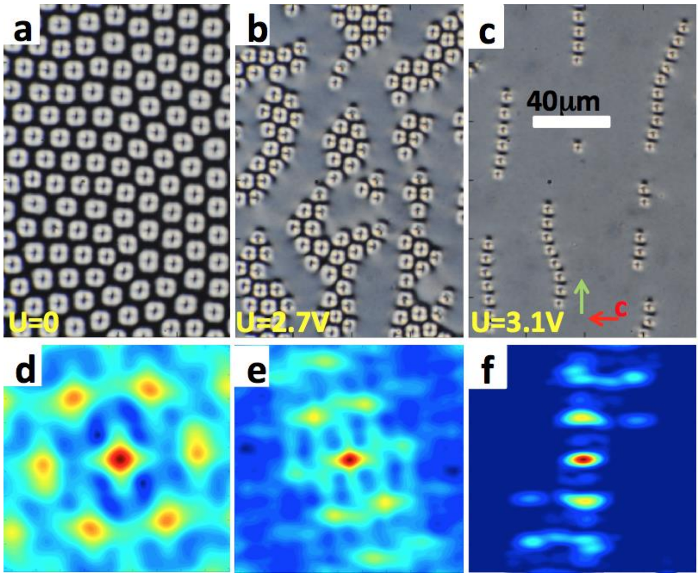

Supplementary Figure 2 | Reconfigurable self-assembly of hopfion-based topological particles. a-**c**, polarizing optical micrographs corresponding to the ones shown in Fig. 2**b**-**d**. **d**-**f**, Auto-correlation of images shown in **a**-**c**, demonstrating field-induced changes in inter-particle distances and transformation between hexagonal and linear-chain self-assemblies of the

topological particles. The intensity in (d-e) increases from blue (lowest) to green, to yellow, and then to red (highest).

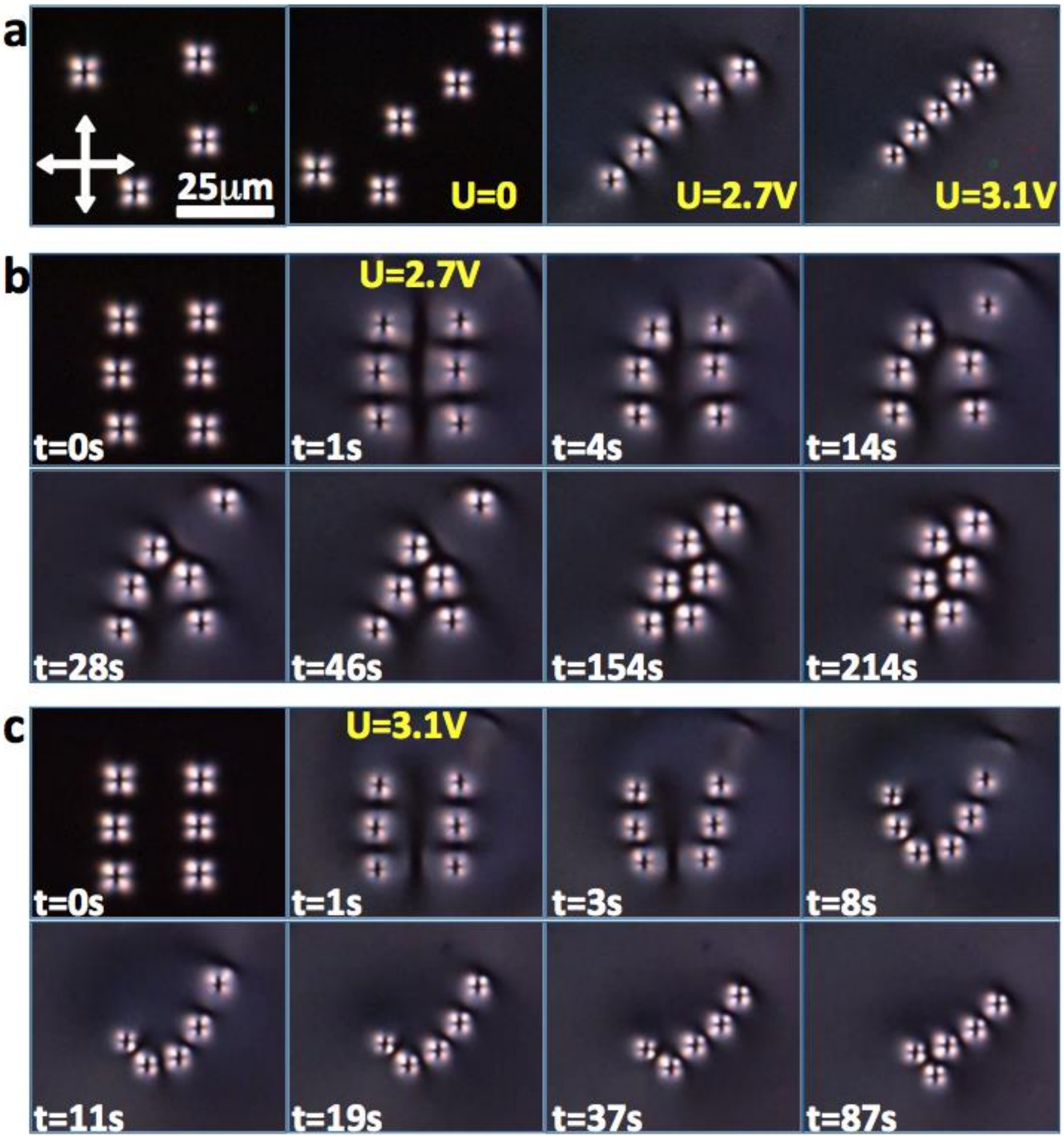

Supplementary Figure 3 | Many-body interactions between hopfion-based particles starting from initial conditions in the forms of arrays subjected to different voltages. a, Laser induced mobile torons (generated at relatively low laser powers ~50 mW) and their interaction at different *U* starting from the same initial conditions. **b**, Structural organization of topological particles versus elapsed time (marked on the images) starting from an initial array of torons and upon application of *U*=2.7V peak to peak, 1 kHz square wave, at which attractive interactions lead to a small-periodicity hexagonal arrangement (Fig. 2c). **c**, Structural organization of topological particles versus elapsed time (marked on the images) starting from an initial array of optically generated torons and upon application of *U*=3.1V peak to peak, 1 kHz square wave, at which strongly anisotropic dipolar interactions lead to a linear-chain self-assembly (Fig. 2d); note that one dipolar

topological particle disappeared (due to the toron-umbilic annihilation) between frames 4 and 5.

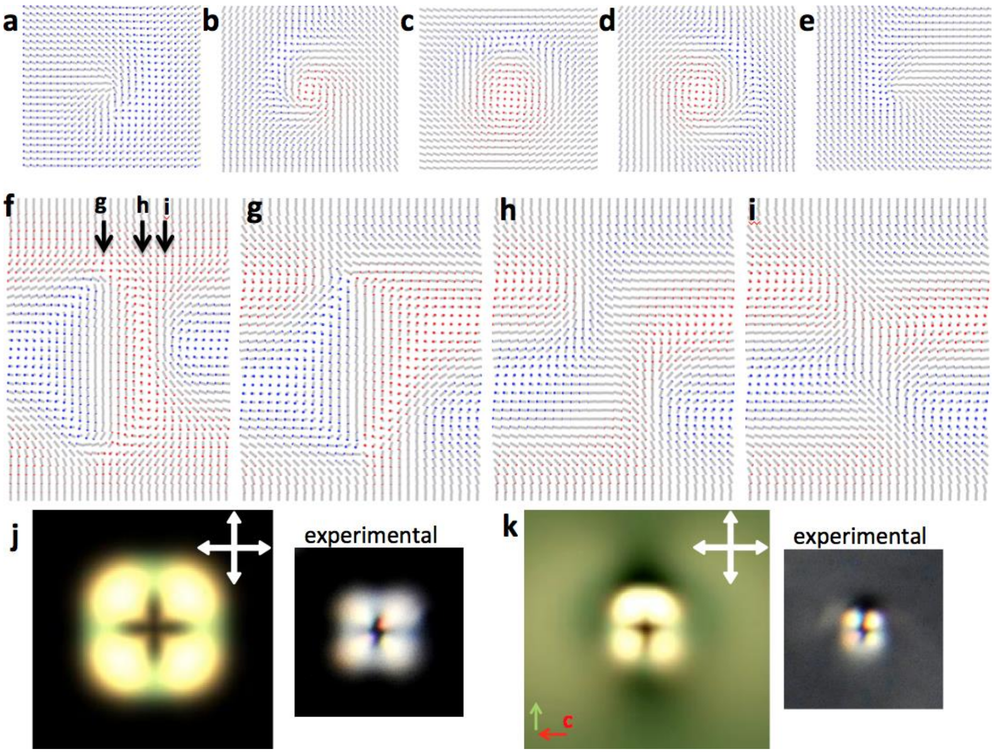

Supplementary Figure 4 | Numerical modeling of the 3D director structure and polarizing optical micrographs of skyrmionic particles in CNLCs. a-e, In-plane cross-sections of a topological particle, whose vertical cross-section is shown in Fig. 1**o**, at five different sample depths while moving from the cell top (**a**) to the cell midplane (**c**) and to (**e**) the bottom part of the cell. **f-i**, Details of director structure of a topological particle with cross-section shown in Fig. 1**o**, which is reproduced here as part (**f**) with labeling of vertical cross-sections orthogonal to it and shown in (**g**-**i**). **j**,**k**, Computer-simulated polarizing optical micrographs corresponding to the director configurations shown in Fig. 1**n**,**o**, respectively; the smaller insets shown to the right of computer-simulated micrographs are the corresponding experimental polarizing optical micrographs.

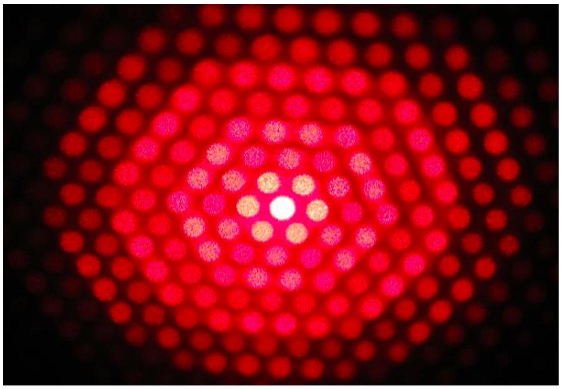

Supplementary Figure 5 | Diffraction pattern obtained using a monodomain hexagonal array of self-assembled topological particles, similar to that shown in Fig. 2a. The pattern was obtained using a HeNe laser beam.

Supplementary Table

Supplementary Table 1. Material parameters of the used nematic ZLI2806.

Properties / LC	K_{11}, pN	K_{22}, pN	K_{33}, pN	K_{24}, pN	$\Delta\varepsilon$
ZLI2806	14.9	7.9	15.4	7.9	-4.8

Supplementary Methods

1. Modeling of structures through minimization of free energy

We use numerical minimization of free energy to obtain the equilibrium and metastable configurations of **n**(**r**) in confined CNLCs. Assuming infinitely strong homeotropic surface anchoring at confining glass plates, the 3D equilibrium **n**(**r**)-structures at different applied electric fields **E** are modeled based on the minimization of bulk free energy $F = F_{elastic} + F_{electric}$ consisting of electric energy $F_{electric} = -(\varepsilon_0 \Delta\varepsilon / 2)\int(\mathbf{E}\bullet\mathbf{n})^2 dV$ and Frank-Oseen elastic energy

$$F_{elastic} = \int\left\{\frac{K_{11}}{2}(\nabla\cdot\mathbf{n})^2 + \frac{K_{22}}{2}[\mathbf{n}\cdot(\nabla\times\mathbf{n}) + \frac{2\pi}{p}]^2 + \frac{K_{33}}{2}[\mathbf{n}\times(\nabla\times\mathbf{n})]^2 - K_{24}[\nabla\cdot[\mathbf{n}(\nabla\cdot\mathbf{n}) + \mathbf{n}\times(\nabla\times\mathbf{n})]]\right\}dV, \quad (1)$$

where $\Delta\varepsilon$<0 is dielectric anisotropy and K_{11}, K_{22}, K_{33}, and K_{24} are elastic constants describing splay, twist, bend and saddle splay deformations, respectively. The saddle-splay constant K_{24} is difficult to measure experimentally[1,2] and is unknown for the used CNLC.[2] We assume K_{24} = K_{22}, similar to previous studies.[1-3] Through numerical modeling, we find that torons in cells with thickness *d* in micrometer range are ground-state structures for $d/p \approx 1$ even when this term is not taken into account (at K_{24} =0), although taking K_{24} = K_{22} further helps to stabilize these skyrmionic field configurations, as we have discussed for torons previously.[3] We therefore set K_{24} = K_{22} while using all other experimentally measured elastic and dielectric constants of the used liquid crystal (Supplementary Table 1).

Minimization of the free energy to find the equilibrium director field is implemented with the relaxation method.[2] Electric free energy is calculated with a static voltage profile. This is a reasonable assumption because the absolute value of the dielectric anisotropy $\Delta\varepsilon$ of the used material is relatively small. Equilibrium 3D-structures of **n**(**r**) have $\delta F / \delta n_i = 0$, where n_i is the projection of the director **n**(**r**) onto the i-axis (i= 1(x), 2(y), 3(z)) and $\delta F / \delta n_i$ are the functional derivatives of the free energy defined as $F = \int f_{total} dV$, with *V* being the volume of the sample. From a numerical point of view, the spatial derivatives of **n**(**r**) are computed using the 2nd order finite difference scheme in a volume broken up into a rectangular computational grid. Periodic boundary conditions are applied along the $\hat{x}$- and $\hat{y}$- directions while fixed homeotropic boundary condition are used along the $\hat{z}$-direction. At each step Δt, the functional derivatives $\delta F / \delta n_i$ and the resulting elementary displacement δn_i defined as $\delta n_i = -\Delta t \dfrac{\delta F}{\delta n_i}$ are computed. The maximum stable time step used in the relaxation routine is determined as $\Delta t = \dfrac{\min(h_i)^2}{2\max(K)}$, where $\min(h_i)$ is the smallest computational grid spacing and $\max(K)$ is the largest elastic constant. The steady state is determined through monitoring the change with respect to time of the spatially averaged functional derivative given by the Lagrange equation. When this value asymptotically approaches zero, the system is assumed to be in a ground state. The

discretization is done on a fairly large grid (119 x 119 x 35), which is important to assure that the minimum-energy **n**(**r**) is indeed a structure localized in space in equilibrium with the surrounding untwisted CNLC or TIC and that the periodic boundary conditions do not introduce artifacts affecting its stability. Using grid spacing of $h_x = h_y = h_z = 0.1$ μm and 35 grid points across the cell gives sample thickness $d = 3.50$ μm, comparable to that used in experiments (although slightly smaller). All presented simulations have been done for $d/p = 1$ and for material parameters of nematic host ZLI2806 provided in the Supplementary Table 1.

For all voltages *U*, the initial director configuration used as a starting point of the minimization procedure described above was a toron director field configuration deduced from the experiments at *U*=0. Additionally, to obtain the equilibrium **n**(**r**)-field configurations at different *U*, the toron was surrounded by TIC configuration for the corresponding *U* relaxed separately;[3] the entire sample volume was then relaxed for the respective *U*. The relaxed director configurations at different voltages and *d*/*p* ratios yield a broad range of **n**(**r**)-structures discussed in details in the main text of this work, which are found to be in agreement with experiments.

2. Laser manipulation and 3D optical imaging

We used an integrated system for simultaneous optical manipulation and 3D imaging, which was built around an inverted microscope IX 81 (Olympus). The holographic optical trapping part of the setup utilizes a reflective, electrically addressed, phase-only spatial light modulator (XY series from Boulder Nonlinear Systems, P512-1064) containing 512 x 512 pixels, each 15 x 15 μm^2 in size. It also employs an Ytterbium-doped fiber laser (YLR-10-1064, IPG Photonics) operating at 1,064 nm. The laser beam is linearly polarized with a Glan-laser polarizer and the linear polarization direction is adjusted with a half-wave retardation plate to optimize the phase modulation efficiency of the spatial light modulator. Before the beam is incident on the spatial light modulator, it is expanded to overfill its active area and then, after being reflected from the modulator, is resized so as to overfill the back aperture of the objective. The spatial

light modulator controls the phase of the beam on a pixel-by-pixel basis according to the computer-generated holographic patterns supplied at a refresh rate of 30 Hz for the entire pixel array. This spatially phase-modulated beam is imaged at the back aperture of the microscope objective, which recreates the 3D spatial trap pattern in the sample. A custom-designed dichroic mirror (obtained from Chroma Technology Corp.) reflects the trapping beam at 1,064 nm while allowing visible light (used for imaging purposes) to transmit through it to the confocal microscopy scanning head and charge-coupled device (CCD) camera.

In both two-photon excitation fluorescence polarizing microscopy (2PEF-PM) and three-photon excitation fluorescence polarizing microscopy (3PEF-PM)[4,5], we have employed a single tunable (680-1080 nm) Ti-Sapphire oscillator (Chameleon Ultra II, Coherent) emitting 140 fs pulses at the repetition rate of 80 MHz. In the 2PEF-PM experiments, we tune the wavelength to 980 nm for the two-photon excitation of the dye BTBP (which was added to some of the samples). The three-photon excitation and the subsequent 3PEF-PM imaging of 5CB-based samples is done without the use of dyes as the chiral agent CB15 molecules within the CNLC strongly fluoresce themselves as a result of the three-photon-absorption-based excitation at 870 nm. The focus of the beam is steered laterally throughout the focal plane in the sample with galvano mirrors. The 2PEF-PM signal from the BTBP dye and 3PEF-PM signal from CB15 molecules is collected in epi-detection mode with a photomultiplier tube (H5784-20, Hamamatsu) and a series of interference filters. Transmission of both white light from a lamp source (Fig. 1e-h, 2a-d,g, 5 and Supplementary Figures 1-3) and monochromatic light (Fig. 4a) through the sample between crossed polarizers is used in polarizing microscopy studies. The use of scanned monochromatic laser light allowed us to simultaneously obtain co-located polarizing optical micrographs and both 2PEF-PM or 3PEF-PM depth-resolved images, an example of which is shown in Fig. 4a,b. We use oil-immersion objectives with high numerical aperture (NA), 60x (NA=1.42) and 100x (NA=1.4), both from Olympus. The same objectives are used for imaging as well as optical trapping. The **n**(**r**) and its azimuthal orientation patterns presented in the main text were obtained through the analysis of Stokes parameters described in details in Ref. 6.

3. Preparation of polymerized CNLC samples

The partially polymerizable cholesteric LC composite was prepared by first mixing 69% of nonreactive negative-dielectric-anisotropy nematic AMLC-001 (from Alpha Micron Inc.) with 30% of a diacrylate nematic (consisting of 12% of RM 82 and 18% of RM 257 obtained from EM Chemicals) and 1% photoinitiator Irgacure 184 (from CIBA Specialty Chemicals), which was then followed by doping this nematic mixture with CB15 (from EM Chemicals) to obtain a cholesteric of pitch *p* equal to 15 μm.[7] The ensuing mixture was first dissolved in dichloromethane to homogenize, heated to 80 °C for one day to remove the solvent through slow evaporation, and then cooled down to obtain a room-temperature CNLC mixture. This CNLC mixture was then used to optically generate and subsequently polymerize at different applied voltages solitonic structures of interest. The cholesteric mixture was infiltrated into LC cells with thickness *d* comparable to *p*. To fabricate the cells, glass substrates with conductive indium tin oxide coatings were spin coated with polyimide SE1211 (Nissan) at 2700 rpm for 30 s and then baked (5 min at 90 °C followed by 1 h at 180 °C) to set strong vertical surface boundary conditions for the LC director. This particular type of samples was used to obtain depth-resolved images similar to that shown in Fig. 1i.

Supplementary References

1. Polak, R.D., Crawford, G.P., Costival, B.C., Doane, J.W. and Zumer, S. Optical determination of the saddle-splay elastic constant K_{24} in nematic liquid crystals. *Phys. Rev. E* **49**, R978 (1994).

2. Anderson, J. E., Watson, P. E. & Bos, P. J. LC3D: Liquid crystal display 3-D director simulator software and technology guide (Artech House, Boston, 2001)

3. Smalyukh, I. I., Lansac, Y., Clark, N. & Trivedi, R. Three-dimensional structure and multistable optical switching of Triple Twist Toron quasiparticles in anisotropic fluids. *Nature Mater.* **9**, 139-145 (2010).

4. Lee, T., Trivedi, R. P. & Smalyukh, I. I. Multimodal nonlinear optical polarizing microscopy of long-range molecular order in liquid crystals. *Opt. Lett.* **35**, 3447-3449 (2010).

5. Trivedi, R. T., Lee, T., Bertness, K. A. & Smalyukh, I. I. Three dimensional optical manipulation and structural imaging of soft materials by use of laser tweezers and multimodal nonlinear microscopy. *Optics Express* **18**, 27658 (2010).

6. Chen, B. G., Ackerman, P. J., Alexander, G. P., Kamien, R. D. & Smalyukh, I. I. Generating the Hopf fibration experimentally in nematic liquid crystals. *Phys. Rev. Lett.* **110**, 237801 (2013).

7. Evans, J. S., Ackerman, P. J., Broer, D. J., van de Lagemaat, J., & Smalyukh, I. I. Optical generation, templating, and polymerization of three-dimensional arrays of liquid-crystal defects decorated by plasmonic nanoparticles. *Phys. Rev. E* **87**, 032503 (2013).
